# Reframing EHR Policy in China: Towards Balanced Implementation and Ethical Foundations

**DOI:** 10.1111/jep.70273

**Published:** 2025-10-14

**Authors:** Shuyuan Chen, Wenhao Huang, Yan Xu, Jie Yang

**Affiliations:** ^1^ School of Management Xuzhou Medical University Xuzhou Jiangsu Province China; ^2^ The Second Clinical Medical College Xuzhou Medical University Xuzhou Jiangsu Province China; ^3^ College of Cell Therapy Drug Industry Xuzhou Medical University Xuzhou Jiangsu Province China

**Keywords:** digital healthcare, electronic health record system, NATO model, policy tool analysis, text data mining

## Abstract

**Objective:**

Electronic Health Record (EHR) systems are a core component of healthcare informatization, playing a crucial role in the development of digital and smart healthcare. This study utilizes policy tool theory to analyse policies related to China's EHR system. By gaining a deep understanding of the mechanisms of policy impact, we believe we can better guide the development direction of EHR systems, optimize resource allocation, and improve system effectiveness and efficiency. Ultimately, this will make a substantial contribution to the construction of healthcare informatization.

**Methods:**

The research employs Christopher Hood's ʻNATOʼ model of policy tools to construct a three‐dimensional analytical framework. A total of 427 relevant policy documents were retrieved and screened from the PKULAW.COM and WWW.GOV.COM databases. NVivo 14 software was used for text mining and quantitative analysis.

**Results:**

The study reveals that authority tools (36.88%) dominate across most policy objectives, particularly in improving healthcare quality and promoting technological innovation. Nodality tools (27.90%) also demonstrate significant influence in enhancing healthcare quality and fostering technological innovation. Organization tools (21.75%) reflect governmental efforts in institutional development and organizational coordination. While economic tools (13.48%) show a lower overall frequency of use, they are applied relatively more in objectives related to rational resource allocation.

**Conclusion:**

Recommendations include optimizing the government's role and balancing the use of authority tools; refining the policy objective system with increased attention to ethical issues; and strengthening the synergistic application of policy tools to enhance policy implementation effectiveness.

## 1 Introduction

Electronic Health Records (EHRs) form core infrastructure in modern healthcare, integrating diverse patient data to enable secure, real‐time access essential for improving care coordination, operational efficiency, and data‐driven research [1, 2]. Since the emergence of seminal systems like the 1960s Massachusetts General Hospital outpatient application, global advancements have focused on expanding EHR capabilities while addressing persistent challenges [3, 4]. China's trajectory demonstrates a notable evolution from adoption to targeted innovation. Initiated by early national commitments in the 1980s and accelerated through comprehensive government policies, infrastructure investment, standardization, platform development, innovation incentives, and workforce training, China achieved near‐universal EHR coverage (99.0%) in tertiary public hospitals by 2022, reaching an average Application Level Grade 4 (on an 8‐grade scale).

Despite this progress, notable disparities persist relative to mature systems in developed countries, particularly in functional sophistication, data quality, interoperability, and privacy safeguards [5, 6]. A critical barrier to achieving higher standards is inadequate policy effectiveness, though rigorous impact analysis remains underdeveloped—especially in large, rapidly digitizing health systems like China's. Research on EHR systems internationally reflects a broadening scope and increasing depth, encompassing diverse aspects such as system advantages, potential challenges, technological innovation, and implementation strategies. Agniel et al. highlighted that EHR data may contain biases arising from internal healthcare system processes, emphasizing the necessity for caution in interpreting and utilizing this data [7, 8]. This finding informs ongoing investigations into EHR data accuracy and reliability. Concurrently, the rapid advancement of artificial intelligence (AI) technologies has positioned the integration of EHR and AI as a prominent research focus. This convergence facilitates novel opportunities for clinical research and decision support [9]. Murali et al.'s work on constructing medical knowledge graphs from EHR data further illustrates the significant potential within this domain [10]. Researchers recognize, however, that ensuring health equity and safeguarding patient privacy remain critical considerations when advancing such technological applications [11, 12]. To optimize EHR benefits while mitigating associated risks, multiple strategies have been proposed. These include employing safety briefings to proactively identify and address EHR‐related safety concerns [13]. Further, studies advocate for EHR system refinements to better capture and encode the cognitive processes underlying clinical decision‐making [14], reflecting sustained efforts to enhance system functionality. Investigations also address EHR deployment in specific contexts. Research examining EHR adoption in rural settings [15] and assessing the impact of EHR systems on hospital clinical performance [16] provides valuable insights for refining implementation strategies and guiding broader dissemination efforts. Despite progress, the implementation and utilization of EHR systems present persistent challenges. This deficit stems from two intertwined research gaps: (1) the absence of robust theoretical frameworks integrating public policy concepts to systematically dissect EHR policy formulation, implementation, and impact mechanisms; and (2) the scarcity of quantitative methodologies for longitudinal, objective evaluation of policy outcomes. Without addressing these, developing evidence‐based refinements to optimize EHR systems and maximize healthcare benefits is significantly constrained.

Directly confronting this need, this study introduces formal public policy instrument theory to EHR research. This theoretical innovation enables a novel multidimensional framework to systematically characterize policy attributes, implementation pathways, and impact mechanisms within China's EHR landscape. Addressing the methodological gap, we rigorously apply quantitative techniques to conduct a comprehensive longitudinal assessment of China's EHR policy evolution. This yields objective metrics to evaluate outcomes, identify drivers of implementation success or failure, and diagnose root causes of challenges during this critical developmental stage. Further leveraging this integrated approach, the research delineates the external characteristics and evolutionary trajectory of China's EHR policies, clarifying historical patterns, inflection points, and primary development drivers to contextualize past decisions and inform future strategy.

Integrating this framework with advanced quantitative evaluation and evolutionary analysis provides a holistic analytical foundation. Findings offer empirically grounded insights to guide targeted policy interventions, optimizing EHR systems for enhanced healthcare quality, efficiency, and equity. Beyond China, the methodology establishes a transferable paradigm for evaluating EHR policies in emerging economies, facilitating global knowledge exchange and accelerating health information system advancement.

**Figure 1:**
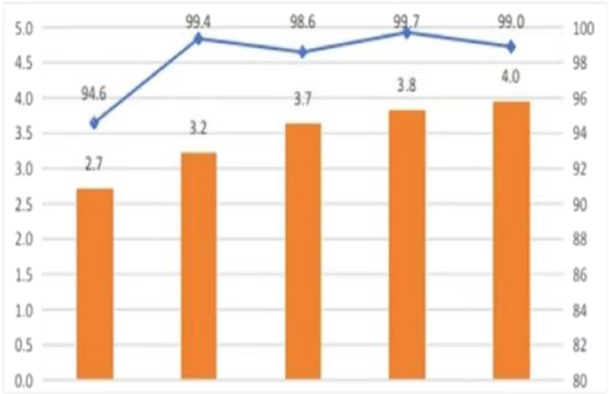
The histogram shows the application level of the electronic medical record system, and the line drawing of the electronic medical record system application level is graded and evaluates the participation rate.

The article is structured as follows: Section Methods, 2 details the methodology. Section Results, 3 introduces and constructs the analytical framework. Section Conclusion, 4 presents results. Section 5 is the conclusion. Section 6 is the discussion section. Section 7 identifies future research directions (Figure 1).

## 2 Materials and Methods

### 2.1 Data Sources

Accounting for substantial regional heterogeneity across China, this investigation centres exclusively on centrally issued policy documents. The search protocol incorporated two authoritative government‐sanctioned databases: PKULAW.COM and the WWW.GOV.CN portal. We employed Chinese‐language Boolean operators combining the core terminology ʻelectronic health record systemʼ, ʻelectronic medical recordʼ, and ʻelectronic health fileʼ to identify relevant policy instruments. This strategy yielded an initial collection of 7868 candidate documents.

To ensure comprehensive coverage of national‐level EHR policies while maintaining analytical precision, a sequential refinement process was implemented. This involved (1) thematic verification of EHR policy relevance, (2) confirmation of issuance by central government authorities, and (3) expert assessment of each document's substantial impact on China's EHR framework evolution. Following this multistage validation, 427 policy instruments were retained for formal analysis (representative samples summarized in Table 1). This systematically refined corpus enables rigorous assessment of policy instrument deployment patterns within China's nationally coordinated EHR implementation landscape.

**Table 1:** Selected policy documents are presented in part.

The name of the file	Issuing authority	Date of posting
Circular of the General Office of the State Council on Printing and Distributing the Implementation Plan for Major Projects for the Revitalization and Development of Traditional Chinese Medicine	General Office of the State Council	2023.02.10
Notice of the General Office of the State Council on Printing and Distributing the 14th Five‐Year Plan for National Health	General Office of the State Council	2022.04.27
Opinions of the General Office of the State Council on Strengthening the Performance Appraisal of Tertiary Public Hospitals	General Office of the State Council	2019.01.16
Notice of the National Health Commission on Further Promoting the Informatization Construction of Medical Institutions with Electronic Medical Records as the Core	National Health Commission	2018.08.22
The National Health and Family Planning Commission issued a notice on 57 health industry standards, including the ʻElectronic Medical Record Sharing Column Specification Part 1: Medical Record Summaryʼ	National Health and Family Planning Commission	2016.08.23
Notice of the General Office of the Ministry of Health on Printing and Distributing the ʻTrial Implementation of Grading Evaluation Methods and Standards for the Functional Application Level of Electronic Medical Record Systemʼ	Ministry of Health (revoked)	2011.10.24

### 2.2 Methodology

**Figure 2:**
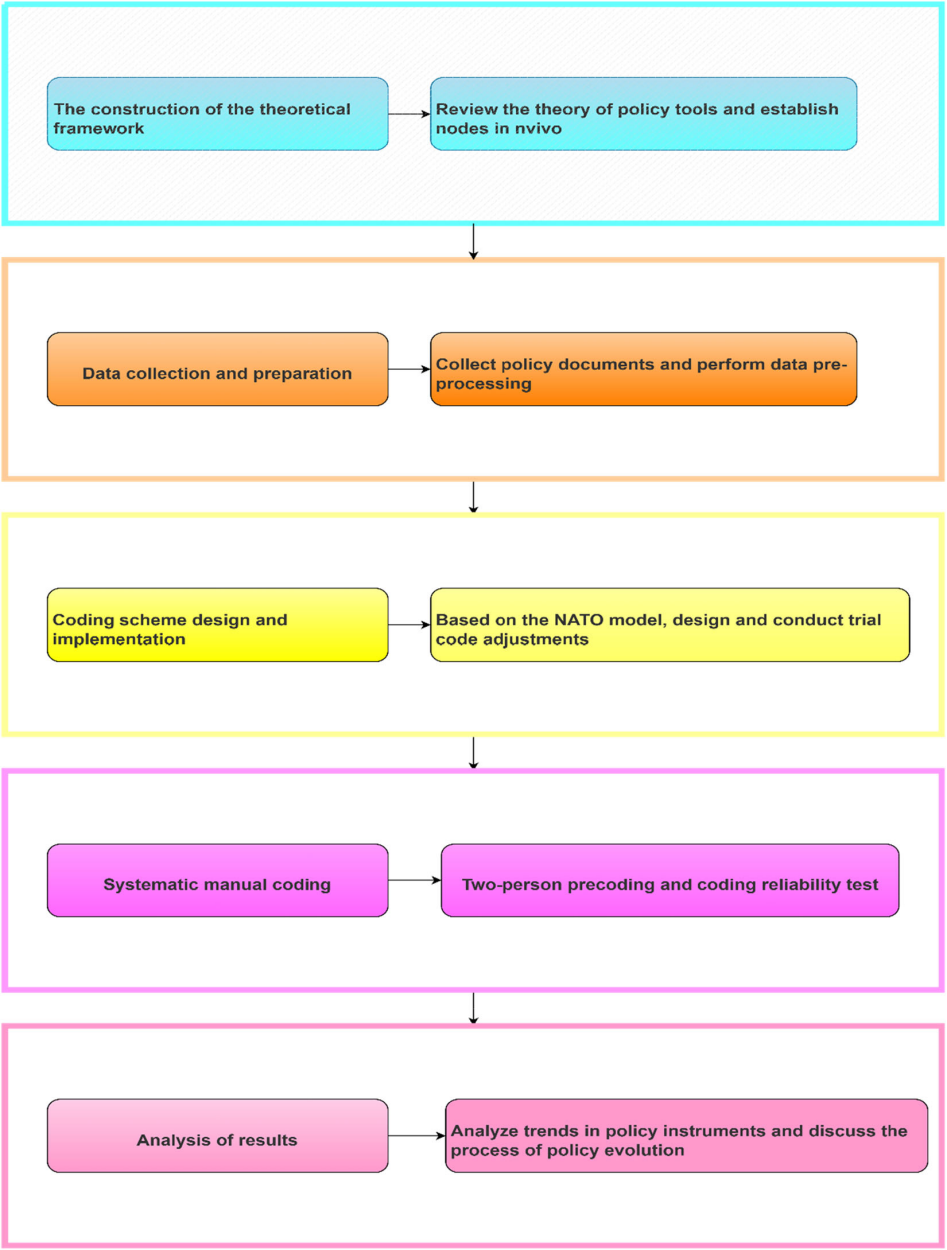
Research process.

The research workflow is delineated in Figure 2. This study employed systematic textual analysis to identify and classify policy instruments within central government documents. Two pretrained researchers independently executed content coding following protocol standardization. To ensure intercoder reliability, 20% of the policy clauses were randomly selected for parallel coding [17], and Cohen's kappa (*κ*) coefficient was calculated [18].

Discrepant coding outcomes were adjudicated by the lead investigator to resolve interpretive ambiguities. Upon refining coding protocols, the remaining 80% of clauses were processed by a single analyst. All *κ* values exceeded the 0.80 threshold, indicating robust analytical reliability. Subsequent textual analysis incorporated frequency enumeration and temporal trend examination. This methodological integration enabled:

1. Quantification of policy instrument deployment dynamics.
2. Identification of qualitative evolutionary patterns within China's EHR policy landscape.
3. Multidimensional examination of developmental trajectories.

The synthesis of traditional policy analysis with computational text interrogation facilitated comprehensive elucidation of policy evolution nuances unattainable through singular methodological approaches.

## 3 Construction of a Three‐Dimensional Framework: Policy Instruments, Policy Evolution, and Policy Objectives

**Figure 3:**
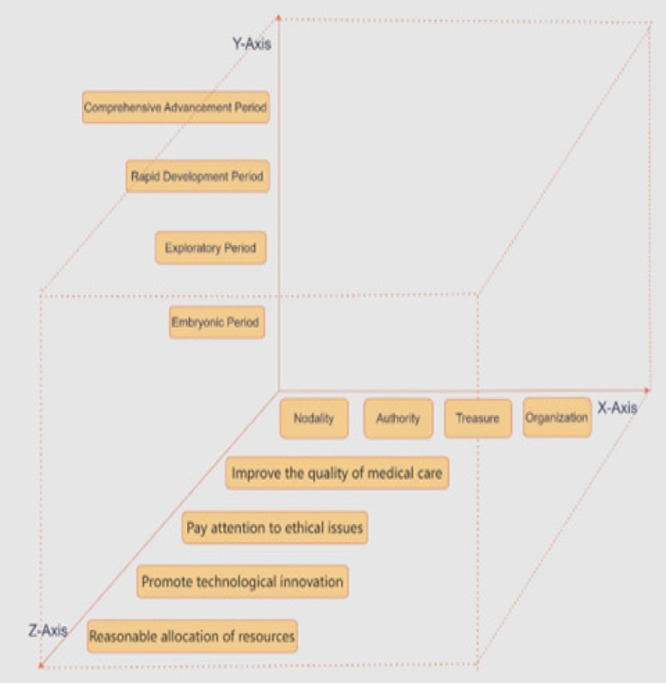
Three‐dimensional analysis framework.

In the study of EHR system policies, this study proposes a three‐dimensional analytical framework (see Figure 3) to comprehensively understand the complexity of policy formulation and implementation. This framework encompasses three dimensions: policy instruments, policy evolution, and policy objectives, aiming to provide a multifaceted, systematic approach to analysing the formulation, implementation, and effectiveness of EHR policies. This multidimensional framework enables an in‐depth exploration of various aspects of EHR policies and reveals the interactions and influences among these dimensions, thereby offering more comprehensive and profound insights for policymakers and researchers.

### 3.1 X‐Dimension: Policy Instruments

Policy instruments are crucial means used by governments to influence policy processes and achieve established goals [19]. They serve as both methods and pathways for governance and as important bridges connecting policy objectives with actual outcomes. Scholars have proposed various classification methods for policy instruments, reflecting the complexity and diversity in this field. McDonell and Elmore proposed a fundamental four‐fold classification, categorizing government tools into mandates, inducements, capacity‐building, and system‐changing instruments. This classification provides an initial framework for understanding basic types of policy instruments [20]. Building on this, Linder and Peters proposed a more detailed and diversified classification method, including mandates, grants, regulations, taxes, exhortation, authority, and contracts [21, 22]. This refined classification aids in a more comprehensive grasp of specific forms of policy instruments. From another perspective, Howlett and Ramesh categorized policy instruments into voluntary, mixed, and mandatory tools based on the degree of government intervention and resultant coerciveness [23]. This classification method emphasizes the degree of coercion in implementing policy instruments, offering a new perspective for understanding their practical operation. Among these classification methods, this study adopts the ʻNATOʼ model of policy instruments proposed by British scholar Christopher Hood as its theoretical foundation [24]. This model categorizes policy instruments into four types: Nodality, Authority, Treasure, and Organization. Hood's model provides a comprehensive framework that can effectively encompass and analyse various types of policy instruments while offering systematic guidance for policy formulation and implementation.

### 3.2 Y‐Dimension: Policy Evolution

The policy evolution dimension focuses on the changes in EHR policies over time. This dimension not only reflects how policymakers adjust and optimize policies based on implementation effects, technological advances, and changing social needs but also showcases important milestones and turning points in the entire medical informatization process. Through in‐depth analysis of policy evolution, we can more comprehensively grasp the developmental trajectory of EHR policies, gain insight into the inherent logic of policy formulation, and provide valuable references for future policy‐making [25]. Furthermore, the dynamic perspective provided by the policy evolution dimension helps us understand the continuity and transformative nature of policy‐making [26]. Continuity is reflected in how policymakers gradually improve and optimize EHR systems on existing foundations, ensuring policy stability and predictability. Transformative aspects are reflected in how policymakers respond to emerging technologies, changing social needs, and unexpected events through policy innovation to address new issues. This balance between continuity and transformation is crucial for the long‐term success of EHR policies. Simultaneously, policy evolution analysis can reveal potential challenges and opportunities in the policy evolution process. Challenges may include asynchrony between technological updates and policy adjustments, compatibility issues between new and old systems, and conflicts between privacy protection and data utilization [27, 28]. Opportunities may manifest in efficiency improvements brought by new technologies, in‐depth mining of data value, and innovation in healthcare service models [29, 30, 31]. By identifying these challenges and opportunities, policymakers can more specifically formulate responsive strategies to promote continuous optimization of EHR policies.

### 3.3 Z‐Dimension: Policy Objectives

Policy objectives are the intended purposes and effects that decision‐makers aim to achieve through policy implementation, with different types of policy instruments corresponding to different policy objectives [32]. In EHR system construction policies, the objective dimension is closely related to the policy evolution dimension, jointly forming the core of policy formulation and implementation. It reflects the long‐term vision of EHR system construction, balancing various stakeholder demands and overall social interests. Notably, EHR policy objectives constitute a multilayered, dynamic, and complex system. At the macro level, objectives may include improving overall healthcare service quality, promoting effective allocation of medical resources, and advancing medical informatization [33]. At the meso level, objectives may involve improving information sharing among medical institutions, enhancing diagnostic efficiency, and reducing medical error rates. At the micro level, objectives may include enhancing patient experience, protecting personal privacy, and promoting effective utilization of medical data [34, 35]. These objectives at different levels are interrelated, collectively forming a comprehensive objective system.

## 4 Results

### 4.1 Descriptive Analysis of China's EHR System Policy Instruments (See Table 2, Table 2 Refers to the Location of the Sentence, e.g., 1‐2‐3 Refers to the Third Sentence of the Second Paragraph of the First Document)

**Table 2:** A descriptive analysis of the policy tools of China's electronic health record system.

Policy tools	Prominent reference nodes (some displays)	Location information	Total (percentage)
Nodality tools	The National Health Commission continues to publish and update standards related to electronic health records, such as the National Medical and Health Information Interconnection Standard System	1—2—4	354 (27.90%)
	Through the official channels of the National Health Commission, progress reports on the construction of electronic health records are regularly released to guide public opinion and industry attention	42—3—4	
	Establish a national level of medical and health information and promote regional level and interconnection	296—6—2	
	Organize and carry out Internet + medical and health demonstration projects, set benchmarks, and promote and expand experience	243—2—8	
	…	…	
Authority tools	Implement the (Opinions of the General Office of the State Council on Promoting the Development of Internet + Medical Health) to clarify the development direction of electronic health records	343—1—6	468 (36.885%)
	The electronic health record should be built as an important indicator for hospital grade evaluation	167—3—3	
	Formulate the Security Law and the Data Security Law to regulate the collection, storage and use of medical and health data	272—7—2	
	Refers to the implementation of the electronic medical record grading and evaluation system, and urges medical institutions to improve the level of application of electronic medical records	35—3—7	
	…	…	
Treasure tools	The central government has set up special funds to support the informatization of primary medical and health institutions, including electronic health record systems	305—7—5	171 (13.48%)
	The application of electronic health records will be linked to medical insurance payment, and medical institutions will be encouraged to improve the level of informatization	235—5—7	
	Through government procurement, the unified construction of regional bioelectronic health equalization will reduce the construction cost of individual medical institutions	14—1—4	
	Additional subsidies will be given to the construction of electronic health record systems for medical institutions in Guanyin areas to narrow regional gaps	397—3—5	
	…	…	
Organization tools	A specialized informatization department is established under the National Health Commission to coordinate the advancement of medical informatization construction, including electronic health records.	32—7—4	276 (21.75%)
	A National Health Medical Big Data Center is established to coordinate the collection, storage, and utilization of national health medical data.	276—2—7	
	An interdepartmental coordination mechanism is established, such as the National Health Commission, Development and Reform Commission, and Ministry of Industry and Information Technology, to jointly advance the ʻInternet Plusʼ healthcare initiative.	120—4—6	
	Medical health informatization leading groups are established in each province and city to oversee the implementation and supervision of electronic health record systems in their respective regions	102—3—2	
	…	…	
			1269 (100%)

Nodality Tools (27.90%) primarily focus on information dissemination and standard‐setting. The National Health Commission (NHC) plays a central role in this area, continuously updating and releasing EHR‐related standards such as the ʻNational Medical Health Information Interoperability Standard Systemʼ to ensure consistency and compatibility across different medical institutions and regions. This standardized approach has significantly promoted nationwide data interoperability and system compatibility. However, achieving complete interoperability across China remains challenging, given the complexity of the Chinese healthcare system and regional disparities.

Authority Tools constitute the largest proportion (36.88%), relying mainly on legislation and regulatory measures to guide EHR development. Key initiatives include implementing the ʻState Council Office's Opinion on Promoting the Development of “Internet + Healthcare”ʼ, incorporating EHR construction into hospital information system (HIS) evaluation standards, and enacting the ʻCybersecurity Lawʼ and ʻData Security Lawʼ. These measures provide a robust legal framework for collecting, storing, and using healthcare data, enhancing public trust. However, legal and regulatory updates may lag behind technological advancements, and balancing data sharing with privacy protection remains challenging in practical implementation [36, 37].

Treasure Tools account for the smallest proportion (13.48%) but play a crucial role in incentivizing medical institutions to adopt EHRs. A report by the Healthcare Information and Management Systems Society (HIMSS) further corroborates the economic benefits of EHR systems. This report indicates that nationwide implementation of interconnected EHR systems would result in annual savings of $78 billion in healthcare expenditures—equivalent to approximately 4% of total national healthcare spending [38]. The current low proportion of treasure tools in promoting EHR systems may lead to potential issues. First, insufficient economic incentives might fail to fully mobilize market forces and private sector engagement, potentially affecting the speed and quality of EHR system adoption. Second, overreliance on government subsidies may struggle to sustainably support long‐term maintenance and upgrades of EHR systems, potentially impacting their sustainable development.

Organization Tools (21.75%) focus on creating institutional structures to oversee and coordinate EHR implementation. Key measures include establishing a dedicated informatization department within the NHC, creating a national health and medical big data centre, and setting up cross‐departmental coordination mechanisms. These measures help to coordinate the implementation of EHR systems and improve policy execution efficiency. However, cross‐departmental coordination may face challenges such as conflicts of interest and information barriers [39], while overly centralized decision‐making mechanisms might overlook the practical needs of grassroots and clinical frontlines.

Comprehensive assessment indicates that China's policy instrument system for EHR is overall comprehensive and systematic. The diversified policy tools can promote EHR system development from different angles, facilitating responses to complex medical informatization challenges. The predominance of authority and nodality tools suggests that China has adopted a top‐down, standards‐oriented approach to EHR implementation, which helps ensure nationwide consistency but may face flexibility issues. Additionally, by organizing ʻInternet + Healthcareʼ demonstration projects, China is promoting the dissemination of innovative practices. However, the policy instrument system may need to incorporate more flexibility to rapidly respond to opportunities and challenges brought by new technologies such as AI and blockchain.

### 4.2 Distribution of Policy Instruments From a Policy Evolution Perspective (X–Y Dimensional Cross‐Analysis)

**Figure 4:**
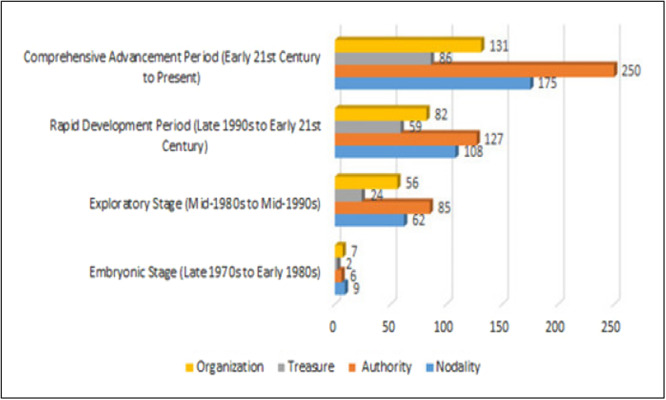
Policy tool distribution from the perspective of policy evolution (X–Y dimension cross‐analysis).

The distribution of policy instruments from a policy evolution perspective (X–Y dimensional cross‐analysis) provides a unique insight into the development trajectory of China's EHR system. By comparing China's development stages with those of developed countries and analysing the utilization of policy instruments, we can clearly observe the evolution of China's EHR policies and the changes in policy instrument usage throughout this process (see Table 3, Figure 4).

**Table 3:** Policy tool distribution from the perspective of policy evolution (X–Y dimension cross‐analysis).

X–Y dimension cross‐analysis	Nodality tools	Authority tools	Treasure tools	Organization tools	Total (percentage)
Nursery stage (late 1970s to early 1980s)	9	6	2	7	24 (1.89%)
Exploration stage (mid‐1980s to mid‐1990s)	62	85	24	56	227 (17.89%)
Rapid development stage (late 1990s to early 2000s)	108	127	59	82	376 (29.63%)
Full promotion stage (early 21st century to present)	175	250	86	131	642 (50.59%)
Total	354 (27.90%)	468 (36.88％)	171 (13.48%)	276 (21.75％)	1269 (100%)

#### 4.2.1 Embryonic Stage (Late 1970s to Early 1980s)

After China's reform and opening up in 1978, the construction of medical informatization began to attract attention. It provides an opportunity for the introduction of foreign advanced technology and equipment. The total number of policy instruments used in this stage was only 24 (accounting for 1.89% of the total during the study period), reflecting that the policy system was in the initial exploration stage. Among them, node tools (9, 37.5%) and organizational tools (7, 29.2%) dominated, indicating that the government mainly promoted informatization by advocating exchanges and learning among medical institutions and organizing the introduction of advanced equipment. In contrast, authoritative tools (6, 25%) and financial securities tools (2, 8.3%) are less used, indicating that a systematic management system has not yet been formed, and financial support is weak. At the same time, developed countries in the United States and Europe have widely used electronic medical record systems in medical institutions (such as the VistA system in the United States) [40, 41, 42]. This highlights the significant generational gap between the level of medical informatization between China and foreign countries at that time, and also lays the foundation for China's subsequent catch‐up development.

#### 4.2.2 Exploratory Stage (Mid‐1980s to Mid‐1990s)

This phase witnessed a primary focus on HIS implementation within China's medical informatization landscape. These initial HIS platforms emphasized administrative functions, offering limited clinical data processing capabilities. Policy engagement intensified significantly during this stage. The documented policy instrument count rose sharply to 227, constituting 17.89% of the total studied, indicating heightened governmental priority. A notable shift occurred in instrument distribution: authority tools (85, 37.4%) superseded nodality tools (62, 27.3%) as the dominant category, reflecting a strategic shift towards guiding development through norms and standards. Organization tools remained prominent (56, 24.7%), while treasure tools, despite increasing to 24 (10.6%), remained comparatively underutilized. Concurrently, developed nations advanced the exploration of comprehensive EHR systems. Influential developments included the US Institute of Medicine's pivotal 1997 report ʻThe Computer‐Based Patient Recordʼ [43] and the initiation of national EHR programmes in the early 1990s [44]. This comparative analysis highlights a lag in China's EHR development trajectory during this period, while concurrently revealing valuable learning opportunities that informed its subsequent advancement.

#### 4.2.3 Rapid Development Period (Late 1990s to Early 21st Century)

This phase marked a significant acceleration in China's EHR system development, reflecting substantially increased governmental priority. Key policy drivers included the Ministry of Health's 1999 Basic Functional Specifications for Hospital Information Systems and the launch of the national Golden Health Project in 2001. Policy instrument deployment peaked at 376 (29.63% of the study period total), underpinning this rapid policy growth. Authority tools remained dominant (127, 33.8%), confirming continued reliance on regulation for EHR advancement. Notably, nodality tool usage surged (108, 28.7%), indicating an enhanced governmental strategy to foster institutional collaboration and knowledge sharing. Concurrent increases occurred in both organization (82, 21.8%) and treasure tools (59, 15.7%), demonstrating stronger commitments to organizational implementation and financial investment. Development remained geographically uneven, however, predominantly concentrated within major urban hospitals while lagging significantly in rural and primary care settings—highlighting persistent implementation disparities. Comparatively, developed nations shifted focus towards addressing EHR interoperability and standardization challenges during this era, exemplified by US initiatives for nationwide electronic records under the Bush administration and the EU's eHealth Action Plan [45]. This divergence underscores the imperative for China to incorporate greater comprehensiveness and foresight in future policy frameworks.

#### 4.2.4 Comprehensive Advancement Period (Early 21st Century to Present)

The early 21st century witnessed China's concerted governmental efforts to drive medical informatization, propelling EHR system development into a phase of comprehensive advancement. This evolution was marked by the release of pivotal policy documents, notably the Opinions on Deepening the Reform of the Medical and Health System (2009), Basic Architecture and Data Standards for Electronic Medical Records (Trial) (2010), and Evaluation Standards for Electronic Medical Record System Application Level (Trial) (2018). Policy instrument deployment surged to 642 instances (50.59% of the total study period), reflecting the intensified depth and breadth of EHR policy. Authority tools (AU) remained predominant (250, 38.9%), signifying an enhanced strategy centred on strengthening regulatory frameworks and standardization. Nodality tool (NOD) usage increased substantially (175, 27.3%), highlighting a greater governmental focus on fostering institutional collaboration and health information exchange. Significant concurrent growth was also observed in organization tools (ORG, 131, 20.4%) and treasure tools (TR, 86, 13.4%), indicating reinforced commitments to systemic implementation and financial investment. Collectively, the trajectory of China's EHR policies reveals a distinct progression: from singular, simplistic, and localized initiatives towards multifaceted, sophisticated, and comprehensive systems. Instrumental application has diversified and refined considerably, with the cumulative count escalating from 24 (embryonic stage) to 642. AU consistently dominated (468 total, 36.88%), closely followed by NOD (354, 27.90%), with ORG (21.75%) and TR (13.48%) comprising the remainder (Figure 4). Notwithstanding the considerable advancements achieved in EHR system construction, a performance gap relative to leading developed nations persists. Future policymakers should prioritize optimizing instrument combinations, with particular emphasis on strengthening TR deployment, while actively leveraging synergistic effects across tool types. This strategic approach is crucial for navigating the challenges and opportunities presented by emerging technologies, enabling further maturation of China's EHR systems.

### 4.3 Impact of Policy Instruments From Different Policy Objective Perspectives (X–Z Dimensional Cross‐Analysis)

Cross‐dimensional analysis examining policy instruments (Z‐dimension) against policy objectives (X‐dimension) revealed distinct patterns in healthcare policy implementation effectiveness (Table 4). The objective ʻImproving healthcare qualityʼ predominated, representing 41.37% (*n* = 525) of all objective citations, reflecting its central priority for policymakers, followed by ʻPromoting technological innovationʼ (28.84%, *n* = 366) and ʻRational resource allocationʼ (24.35%, *n* = 309). Notably, ʻAddressing ethical issuesʼ received markedly less emphasis (4.18%, *n* = 53), suggesting a relative gap despite ongoing technological advancements. Authority tools were the most extensively utilized instrument class across multiple objectives, particularly for quality improvement (*n* = 237) and innovation promotion (*n* = 148), demonstrating the continued primacy of regulatory and legislative approaches in guiding healthcare development. However, this heavy reliance on authority instruments may constrain policy flexibility. For resource allocation objectives, organizational (*n* = 103) and treasure tools (*n* = 98) were more frequently deployed, indicating a practical shift towards structural adjustments and economic incentives beyond purely administrative measures. Nodality tools demonstrated significant application in quality improvement (*n* = 186) and innovation promotion (*n* = 91), consistent with the growing integration of information technology in healthcare systems. Although treasure tools exhibited narrower overall application, their substantive use in resource allocation contexts signalled recognition of fiscal mechanisms' role in optimizing resource flows. The current instrument mix for resource allocation displayed relative balance but warrants further optimization towards integrated approaches combining treasure, organizational, and authority tools to establish more efficient and equitable resource allocation mechanisms. Critically, the substantial underrepresentation of ethical considerations necessitates focused attention [46]; strengthening ethical frameworks through instruments like enhanced data protection legislation, formal ethics education integration, and transparent decision‐making protocols is imperative alongside technological and efficiency pursuits [47].

**Table 4:** The impact of policy instruments from the perspective of different policy objectives (cross‐analysis of X–Z dimensions).

X–Z dimension cross‐analysis	Nodality tools	Authority tools	Treasure tools	Organization tools	Total (percentage)
Improving Healthcare Quality	186	237	28	74	525 (41.37%)
Focusing on Ethical Issues	23	20	4	6	53 (4.18%)
Promoting Technological Innovation	91	148	41	86	366 (28.84%)
Rational allocation of resources	48	60	98	103	309 (24.35%)
Unresponsive reference node	6	3	0	7	16 (1.26%)
Total	354 (27.90%)	468 (36.88%)	171 (13.48%)	276 (21.75%)	1269 (100%)

## 5 Conclusion

This study introduced public policy theory, applying Christopher Hood's ʻNATOʼ model of policy instruments to analyse EHR system policies. By constructing a three‐dimensional analytical framework, we comprehensively evaluated the effects and impacts of China's EHR system policies. The research findings reveal several key insights:

### 5.1 Optimizing Government Role: Balancing the Use of Authority Tools

Our analysis reveals a dominant reliance on authority‐based instruments across core EHR policy objectives, particularly healthcare quality improvement and technological innovation. This underscores the government's central implementation role [48]. However, an overreliance on authority tools necessitates a rebalancing towards complementary policy instruments to enhance overall efficacy. Therefore, while increasing the use of authority tools, we recommend:

a. Maintaining balance among policy instruments [49]: The increase in authority tools should not lead to a weakening of other types of instruments. Instead, an optimal balance should be sought among various tools, creating complementary and synergistic effects. For example, authority tools can be combined with treasure tools, implementing regulations alongside fiscal incentives to ensure both policy enforcement and strong economic motivation.
b. Enhancing policy precision [50]: When formulating and implementing authoritative policies, the actual situations of different regions and levels of medical institutions should be fully considered, avoiding a ʻone‐size‐fits‐allʼ approach. A strategy of classified guidance and tiered implementation can be adopted, developing differentiated policy measures based on the progress and specific challenges of EHR system construction in various regions.
c. Emphasizing policy flexibility: In the face of rapidly changing healthcare environments and technological innovations [51], authoritative policies also need to maintain a degree of flexibility. We suggest establishing regular assessment and adjustment mechanisms to optimize policy content based on implementation effects and emerging issues. For instance, policy pilots could be set up to test initiatives on a small scale before broader implementation.

### 5.2 Refining Policy Objective System: Enhancing Focus on Ethical Issues

The research reveals that current Chinese healthcare policy objectives primarily focus on improving medical quality, promoting technological innovation, and rational resource allocation. This reflects policymakers' high prioritization of enhancing medical service standards, advancing industry progress, and optimizing resource allocation. This focus has undoubtedly played a positive role in promoting healthcare development. Policymakers' strategic choices are rational, as high‐quality medical services, advanced medical technologies, and efficient resource allocation are cornerstones of building a modern healthcare system. By focusing on these areas, China's healthcare sector has made significant progress over the past few decades, substantially improving public health levels and access to medical services.

As a rapidly developing emerging economy, China's healthcare system faces unique challenges and opportunities. While pursuing improvements in medical quality and technological innovation, we cannot ignore the importance of ethical issues for the long‐term healthy development of the medical system. Given these findings, we recommend better integrating ethical considerations into various policy objectives in future policy formulations. Specifically, this can be approached from the following aspects:

a. Incorporating ethical assessments into medical technology innovation processes: While promoting technological innovation, establish strict ethical review mechanisms to ensure that the application of new technologies does not infringe on patient rights or cause social injustice [52].
b. Strengthening ethical training for medical personnel: Incorporate medical ethics education into mandatory courses and continuing education for medical staff to raise ethical awareness across the entire medical system [53].
c. Establishing interdisciplinary research teams: Encourage collaboration among experts in medicine, ethics, law, sociology, and other disciplines to comprehensively assess the ethical impact of healthcare policies [54, 55].

Ethical considerations are not an obstacle to improving medical quality and technological innovation; rather, they are key factors in ensuring the sustainability and social acceptability of medical development. By placing ethical issues on par with quality, innovation, and resource allocation, we can construct a more comprehensive, just, and resilient healthcare system, truly achieving the ultimate goal of serving the health of all people.

This study demonstrates that optimizing China's EHR policy framework necessitates a dual strategy: (1) achieving a synergistic balance in governmental instrument application, and (2) integrating ethical imperatives directly into core policy objectives [56]. These adjustments are critical for developing effective, resilient, and trustworthy healthcare policies capable of navigating technological advancement while safeguarding fundamental patient rights and societal values. Further research on instrument interaction effects and ethical metric development is warranted.

## 6 Discussion

This study advances health policy research through three significant contributions, culminating in a novel methodology tailored for examining EHR governance. By applying computational text‐mining to a corpus of 427 Chinese EHR governance documents, we establish an unprecedented empirical basis for diagnosing policy efficacy, filling a critical gap within health informatics literature. The systematic quantification of resource‐allocation patterns within this national data set identifies systemic inefficiencies across different levels of governance, providing health administrators with evidence‐based intelligence crucial for service optimization. Furthermore, this analysis crucially exposes how centralized EHR governance architectures inherently generate ethical trade‐offs between operational efficiency and healthcare equity—a tension demanding explicit safeguards during implementation planning. To support robust policy evaluation, the developed NATO benchmarking framework offers a significant methodological advance over static models. This framework facilitates evidence‐based comparisons and accommodates essential contextual variations in local implementation environments and regulations. Collectively, these innovations provide: new instrumentation for objectively assessing policy trade‐offs; governance transparency mapping to pinpoint inefficiencies; and evidence‐backed protocols for mitigating digital health disparities. Consequently, this study delivers immediate value: policymakers gain adaptable frameworks for designing context‐sensitive EHR governance; health systems acquire diagnostic tools to identify resource leakage; and equity advocates secure empirical evidence for advocating ethically grounded technology governance. Until then, the methodology presented here—synthesizing advanced text analytics (NVivo), rigorous systems analysis (resource instrumentation mapping), and ethical governance standards—provides an essential foundation for building equitable and effective next‐generation digital health infrastructures in China and beyond.

## 7 Future Directions

Building on this analysis, critical research priorities emerge to advance EHR policy development and optimization. Sustained monitoring of policy evolution remains paramount for the timely identification of dynamic environmental shifts and emergent trends. Future investigations should employ mixed‐methods approaches to deepen understanding of the complex dynamics underlying policy formulation and implementation. Rigorous evaluation of policy effectiveness is essential, moving beyond metrics of goal attainment to systematically examine implementation barriers, adaptive strategies, and unanticipated consequences. Cross‐national comparative analyses will provide crucial benchmarks for contextualizing and refining domestic frameworks. Concurrently, dedicated exploration of the ethical dimensions—particularly concerning data privacy, security, and equitable access—demands priority [57, 58]. As AI and big data rapidly transform healthcare, proactive research must evaluate their implications for EHR systems and governance, informing anticipatory policies that leverage opportunities while mitigating novel risks [59, 60]. Furthermore, empirical assessment of the comparative efficacy and synergistic potential of diverse policy instruments will enhance the precision and impact of regulatory tool selection.

Collectively, research undertaken through this collective lens will significantly enrich the theoretical foundations for evidence‐based policy refinement and provide actionable guidance for stakeholders, driving the advancement of EHR systems and healthcare innovation.

## Author Contributions

Shuyuan Chen spearheaded the intellectual architecture, with primary responsibility for conceptualizing the interdisciplinary framework, designing the NATO policy benchmarking methodology, and drafting the manuscript. Wenhao Huang conducted systematic data acquisition, including computational text‐mining of EHR policies using NVivo 14.0 and quantitative mapping of resource‐allocation instruments. Yan Xu and Jie Yang led critical revision cycles, enhancing theoretical rigour through stakeholder response modelling and ensuring ethical governance implications were integrated throughout the final manuscript.

## Ethics Statement

This article does not contain any studies with human or animal participants.

## Conflicts of Interest

The authors declare no conflicts of interest.

## Data Availability

Data openly available in a public repository that issues data sets with DOIs. The data that support the findings of this study are openly available in The State Council of the People's Republic of China‌ at https://english.www.gov.cn/ and The Laws & Regulations Database‐Chinalawinfo at https://www.pkulaw.com/.

## References

[1] J. Adler‐Milstein , A. J. Holmgren , P. Kralovecn , C. Worzala , T. Searcy , and V. Patel , “Electronic Health Record Adoption in US Hospitals: The Emergence of a Digital “Advanced Use” Divide,” Journal of the American Medical Informatics Association 24 (2017): 1142–1148, 10.1093/jamia/ocx080. 29016973 PMC7651985

[2] M. O. Kim , E. Coiera , and F. Magrabi , “Problems With Health Information Technology and Their Effects on Care Delivery and Patient Outcomes: A Systematic Review,” Journal of the American Medical Informatics Association 24 (2017): 246–250, 10.1093/jamia/ocw154. 28011595 PMC7651955

[3] M. Bloomrosen , J. Starren , N. M. Lorenzi , J. S. Ash , V. L. Patel , and E. H. Shortliffe , “Anticipating and Addressing the Unintended Consequences of Health IT and Policy: A Report From the AMIA 2009 Health Policy Meeting,” Journal of the American Medical Informatics Association 18 (2011): 82–90, 10.1136/jamia.2010.007567. 21169620 PMC3005876

[4] C. McDonald , G. Schadow , M. Barnes , et al., “Open Source Software in Medical Informatics—Why, How and What,” International Journal of Medical Informatics 69 (2003): 175–184, 10.1016/s1386-5056(02)00104-1. 12810121

[5] H. D. Anderson , K. Kim , M. Zhang , E. Gutierrez , J. Malhotra , and C. Tak , “Assessing Availability of Depression Severity Indicators in Electronic Health Record Data: A Retrospective Study in Two Large Academic Health Care Systems in the United States,” Psychological Services (2025), 10.1037/ser0000969. 40455496

[6] A. L. McGuire , R. Fisher , P. Cusenza , et al., “Confidentiality, Privacy, and Security of Genetic and Genomic Test Information in Electronic Health Records: Points to Consider,” Genetics in Medicine 10 (2008): 495–499, 10.1097/gim.0b013e31817a8aaa. 18580687

[7] D. Agniel , I. S. Kohane , and G. M. Weber , “Biases in Electronic Health Record Data Due to Processes Within the Healthcare System: Retrospective Observational Study,” BMJ 361 (2018): k1479, 10.1136/bmj.k1479. 29712648 PMC5925441

[8] P. L. Elkin , S. Mullin , J. Mardekian , et al., “Using Artificial Intelligence With Natural Language Processing to Combine Electronic Health Record's Structured and Free Text Data to Identify Nonvalvular Atrial Fibrillation to Decrease Strokes and Death: Evaluation and Case‐Control Study,” Journal of Medical Internet Research 23 (2021): e28946, 10.2196/28946. 34751659 PMC8663460

[9] Y. Juhn and H. Liu , “Artificial Intelligence Approaches Using Natural Language Processing to Advance EHR‐Based Clinical Research,” Journal of Allergy and Clinical Immunology 145 (2020): 463–469, 10.1016/j.jaci.2019.12.897. 31883846 PMC7771189

[10] L. Murali , G. Gopakumar , D. M. Viswanathan , and P. Nedungadi , “Towards Electronic Health Record‐Based Medical Knowledge Graph Construction, Completion, and Applications: A Literature Study,” Journal of Biomedical Informatics 143 (2023): 104403, 10.1016/j.jbi.2023.104403. 37230406

[11] T. G. James , M. K. Sullivan , J. D. Butler , and M. M. McKee , “Promoting Health Equity for Deaf Patients Through the Electronic Health Record,” Journal of the American Medical Informatics Association 29 (2021): 213–216, 10.1093/jamia/ocab239. 34741507 PMC8714292

[12] C. Campos‐Castillo and D. L. Anthony , “The Double‐Edged Sword of Electronic Health Records: Implications for Patient Disclosure,” Journal of the American Medical Informatics Association 22 (2015): e130–e140, 10.1136/amiajnl-2014-002804. 25059953 PMC11888334

[13] S. Menon , H. Singh , T. D. Giardina , et al., “Safety Huddles to Proactively Identify and Address Electronic Health Record Safety,” Journal of the American Medical Informatics Association 24 (2017): 261–267, 10.1093/jamia/ocw153. 28031286 PMC5391729

[14] J. J. Cimino , “Putting the “Why” in “EHR”: Capturing and Coding Clinical Cognition,” Journal of the American Medical Informatics Association 26 (2019): 1379–1384, 10.1093/jamia/ocz125. 31407781 PMC6798564

[15] B. E. Whitacre , “Rural EMR Adoption Rates Overtake Those in Urban Areas,” Journal of the American Medical Informatics Association 22 (2015): 399–408, 10.1093/jamia/ocu035. 25665701 PMC8485927

[16] N. Yuan , R. A. Dudley , W. J. Boscardin , and G. A. Lin , “Electronic Health Records Systems and Hospital Clinical Performance: A Study of Nationwide Hospital Data,” Journal of the American Medical Informatics Association 26 (2019): 999–1009, 10.1093/jamia/ocz092. 31233144 PMC7647234

[17] G. Becker , “Creating Comparability Among Reliability Coefficients: The Case of Cronbach Alpha and Cohen Kappa,” Psychological Reports 87 (2000): 1171–1182E, 10.2466/pr0.2000.87.3f.1171. 11272758

[18] M. Chmielewski , L. A. Clark , R. M. Bagby , and D. Watson , “Method Matters: Understanding Diagnostic Reliability in DSM‐IV and DSM‐5,” Journal of Abnormal Psychology 124 (2015): 764–769, 10.1037/abn0000069. 26098046 PMC4573819

[19] J. M. Krawiec , O. M. Piaskowska , P. F. Piesiewicz , and W. Białaszek , “Tools for Public Health Policy: Nudges and Boosts as Active Support of the Law in Special Situations Such as the COVID‐19 Pandemic,” Globalization and Health 17 (2021): 132, 10.1186/s12992-021-00782-5. 34801054 PMC8605446

[20] L. M. McDonnell and R. F. Elmore , “Getting the Job Done: Alternative Policy Instruments,” Educational Evaluation and Policy Analysis 9 (1987): 133–152, 10.3102/01623737009002133.

[21] D. A. Salazar‐Morales , “Sermons, Carrots or Sticks? Explaining Successful Policy Implementation in a Low Performance Institution,” Journal of Education Policy 33 (2017): 457–487, 10.1080/02680939.2017.1378823.

[22] S. H. Linder and B. G. Peters , “Instruments of Government: Perceptions and Contexts,” Journal of Public Policy 9 (2008): 35–58, 10.1017/s0143814x00007960.

[23] A. Perl , M. Howlett , and M. Ramesh , “Policy‐Making and Truthiness: Can Existing Policy Models Cope With Politicized Evidence and Willful Ignorance in a “Post‐Fact” World?,” Policy Sciences 51 (2018): 581–600, 10.1007/s11077-018-9334-4.

[24] C. Hood , H. Rothstein , M. Spackman , W. J. Rees , and R. Baldwin , “Explaining Risk Regulation Regimes: Exploring the ‘Minimal Feasible Response’ Hypothesis,” Health, Risk & Society 1 (1999): 151–166, 10.1080/13698579908407015.

[25] L. Wang , X. Li , Z. Ye , S. Zhang , X. Zhang , and L. Jing , “The Ongoing Impact of Policy Documents on the Pandemic Based on the Framework of the “4Rs” Theory and Policy Tools: In China,” BMC Public Health 25 (2025): 1926, 10.1186/s12889-025-22504-x. 40413485 PMC12102847

[26] C. L. McWilliam , “Continuing Education at the Cutting Edge: Promoting Transformative Knowledge Translation,” Journal of Continuing Education in the Health Professions 27 (2007): 72–79, 10.1002/chp.102. 17576632

[27] M. Wei , M. Todd , A. N. C. Campbell , et al., “Balancing Privacy, Trust, and Equity: Patient Perspectives on Substance Use Disorder Data Sharing,” International Journal of Environmental Research and Public Health 22 (2025): 617, 10.3390/ijerph22040617. 40283841 PMC12027209

[28] L. M. Lee , “Ethics and Subsequent Use of Electronic Health Record Data,” Journal of Biomedical Informatics 71 (2017): 143–146, 10.1016/j.jbi.2017.05.022. 28578074

[29] G. E. Brisson , C. Barnard , P. D. Tyler , D. M. Liebovitz , and K. J. Neely , “A Framework for Tracking Former Patients in the Electronic Health Record Using an Educational Registry,” Journal of General Internal Medicine 33 (2018): 563–566, 10.1007/s11606-017-4278-5. 29302880 PMC5880770

[30] J. Vogel , J. S. Brown , T. Land , R. Platt , and M. Klompas , “MDPHnet: Secure, Distributed Sharing of Electronic Health Record Data for Public Health Surveillance, Evaluation, and Planning,” American Journal of Public Health 104 (2014): 2265–2270, 10.2105/AJPH.2014.302103. 25322301 PMC4232140

[31] G. Massen , H. Whittaker , S. Cook , et al., “Using Routinely Collected Electronic Healthcare Record Data to Investigate Fibrotic Multimorbidity in England,” Clinical Epidemiology 16 (2024): 433–443, 10.2147/CLEP.S463499. 38952572 PMC11215821

[32] S. Morgan , M. McMahon , and D. Greyson , “Balancing Health and Industrial Policy Objectives in the Pharmaceutical Sector: Lessons From Australia,” Health Policy 87 (2008): 133–145, 10.1016/j.healthpol.2008.01.003. 18295927

[33] S. Asthana , A. Gibson , and J. Halliday , “The Medicalisation of Health Inequalities and the English NHS: The Role of Resource Allocation,” Health Economics, Policy and Law 8 (2013): 167–183, 10.1017/S1744133112000126. 22947257

[34] S. Avancha , A. Baxi , and D. Kotz , “Privacy in Mobile Technology for Personal Healthcare,” ACM Computing Surveys 45 (2012): 1–54, 10.1145/2379776.2379779.

[35] E. H. W. Kluge , “Informed Consent and the Security of the Electronic Health Record (EHR): Some Policy Considerations,” International Journal of Medical Informatics 73 (2004): 229–234, 10.1016/j.ijmedinf.2003.11.005. 15066551

[36] Y. B. Choi , K. E. Capitan , J. S. Krause , and M. M. Streeper , “Challenges Associated With Privacy in Health Care Industry: Implementation of HIPAA and the Security Rules,” Journal of Medical Systems 30 (2006): 57–64, 10.1007/s10916-006-7405-0. 16548416

[37] X. Wang , A. Zhang , X. Xie , and X. Ye , “Secure‐Aware and Privacy‐Preserving Electronic Health Record Searching in Cloud Environment,” International Journal of Communication Systems 32 (2019): e3925, 10.1002/dac.3925.

[38] B. Tilahun and F. Fritz , “Comprehensive Evaluation of Electronic Medical Record System Use and User Satisfaction at Five Low‐Resource Setting Hospitals in Ethiopia,” JMIR Medical Informatics 3 (2015): e22, 10.2196/medinform.4106. 26007237 PMC4460264

[39] M. Onal Vural , L. Dahlander , and G. George , “Collaborative Benefits and Coordination Costs: Learning and Capability Development in Science,” Strategic Entrepreneurship Journal 7 (2013): 122–137, 10.1002/sej.1154.

[40] J. H. Garvin , M. Kalsy , C. Brandt , et al., “An Evolving Ecosystem for Natural Language Processing in Department of Veterans Affairs,” Journal of Medical Systems 41 (2017): 32, 10.1007/s10916-016-0681-4. 28050745

[41] T. Custers , J. Hurley , N. S. Klazinga , and A. D. Brown , “Selecting Effective Incentive Structures in Health Care: A Decision Framework to Support Health Care Purchasers in Finding the Right Incentives to Drive Performance,” BMC Health Services Research 8 (2008): 66, 10.1186/1472-6963-8-66. 18371198 PMC2329630

[42] D. Kato , J. Lucas , and D. F. Sittig , “Implementation of a Health Information Technology Safety Classification System in the Veterans Health Administration's Informatics Patient Safety Office,” Journal of the American Medical Informatics Association 31 (2024): 1588–1595, 10.1093/jamia/ocae107. 38758666 PMC11187429

[43] R S. Dick , E. B. Steen , and D. E. Detmer , eds. The Computer‐Based Patient Record: Revised Edition: An Essential Technology for Health Care. National Academies Press, 1997). 25121222

[44] J. Duval Jensen , L. Ledderer , R. Kolbaek , and K. Beedholm , “Fragmented Care Trajectories in Municipal Healthcare: Local Sensemaking of Digital Documentation,” Digit Health 9 (2023): 20552076231180521, 10.1177/20552076231180521. 37312959 PMC10259120

[45] D. W. Simborg , “Promoting Electronic Health Record Adoption. Is It the Correct Focus?,” Journal of the American Medical Informatics Association 15 (2008): 127–129, 10.1197/jamia.M2573. 18096904 PMC2274790

[46] K. Yohannes , M. Målqvist , H. Bradby , Y. Berhane , and S. Herzig van Wees , “Addressing the Needs of Ethiopia's Street Homeless Women of Reproductive Age in the Health and Social Protection Policy: A Qualitative Study,” International Journal for Equity in Health 22 (2023): 80, 10.1186/s12939-023-01874-x. 37143037 PMC10159225

[47] L. S. S. Pinto , I. M. P. Oliveira , E. S. S. Pinto , C. B. C. Leite , A. N. Melo , and M. C. B. R. Deus , “Políticas públicas de proteção à mulher: avaliação do atendimento em saúde de vítimas de violência sexual,” Ciência & Saúde Coletiva 22 (2017): 1501–1508, 10.1590/1413-81232017225.33272016. 28538921

[48] C. A. Onoka , K. Hanson , and J. Hanefeld , “Towards Universal Coverage: A Policy Analysis of the Development of the National Health Insurance Scheme in Nigeria,” Health Policy and Planning 30 (2015): 1105–1117, 10.1093/heapol/czu116. 25339634

[49] S. M. Onstwedder , M. E. Jansen , M. C. Cornel , and T. Rigter , “Policy Guidance for Direct‐to‐Consumer Genetic Testing Services: Framework Development Study,” Journal of Medical Internet Research 26 (2024): e47389, 10.2196/47389. 39018558 PMC11292153

[50] G. J. Y. Hsu , Y.‐H. Lin , and Z.‐Y. Wei , “Competition Policy for Technological Innovation in an Era of Knowledge‐Based Economy,” Knowledge‐Based Systems 21 (2008): 826–832, 10.1016/j.knosys.2008.03.043.

[51] C. Martikalini , “Policy Learning in Collaborative Government: A Framework for Adaptive and Resilient Public Policy,” Edelweiss Applied Science and Technology 8 (2024): 9142–9157, 10.55214/25768484.v8i6.3954.

[52] K. Busche , K. W. Burak , P. Veale , S. Coderre , and K. McLaughlin , “Making Progress in the Ethical Treatment of Medical Trainees,” Advances in Health Sciences Education 21 (2016): 711–718, 10.1007/s10459-015-9617-x. 26092833

[53] G. Perera , A. Holbrook , L. Thabane , G. Foster , and D. J. Willison , “Views on Health Information Sharing and Privacy From Primary Care Practices Using Electronic Medical Records,” International Journal of Medical Informatics 80 (2011): 94–101, 10.1016/j.ijmedinf.2010.11.005. 21167771

[54] S. Bennett , “Clinical Writing of a Therapy in Progress: Ethical Questions and Therapeutic Challenges,” Clinical Social Work Journal 34 (2005): 215–226, 10.1007/s10615-005-0011-7.

[55] E. M. Meslin , S. A. Alpert , A. E. Carroll , J. D. Odell , W. M. Tierney , and P. H. Schwartz , “Giving Patients Granular Control of Personal Health Information: Using an Ethics ‘Points to Consider’ to Inform Informatics System Designers,” International Journal of Medical Informatics 82 (2013): 1136–1143, 10.1016/j.ijmedinf.2013.08.010. 24139626

[56] A. J. O'Brien , B. G. McKenna , and R. R. Kydd , “Compulsory Community Mental Health Treatment: Literature Review,” International Journal of Nursing Studies 46 (2009): 1245–1255, 10.1016/j.ijnurstu.2009.02.006. 19296950

[57] A. Darr , M. I. Harrison , L. Shakked , and N. Shalom , “Physicians' and Nurses' Reactions to Electronic Medical Records: Managerial and Occupational Implications,” Journal of Health Organization and Management 17 (2003): 349–359, 10.1108/14777260310505129. 14628488

[58] O. Ben‐Assuli , “Electronic Health Records, Adoption, Quality of Care, Legal and Privacy Issues and Their Implementation in Emergency Departments,” Health Policy 119 (2015): 287–297, 10.1016/j.healthpol.2014.11.014. 25483873

[59] S. Nerella , S. Bandyopadhyay , J. Zhang , et al., “Transformers and Large Language Models in Healthcare: A Review,” Artificial Intelligence in Medicine 154 (2024): 102900, 10.1016/j.artmed.2024.102900. 38878555 PMC11638972

[60] N. A. Nasarudin , F. Al Jasmi , R. O. Sinnott , et al., “A Review of Deep Learning Models and Online Healthcare Databases for Electronic Health Records and Their Use for Health Prediction,” Artificial Intelligence Review 57 (2024): 249, 10.1007/s10462-024-10876-2.

